# Supplementary material for: Metabolic oscillations on the circadian time scale in Drosophila cells lacking clock genes
Source: Mol Syst Biol. 2018 Aug 6;14(8):e8376. doi: 10.15252/msb.20188376 (PMC6078164; doi:10.15252/msb.20188376)
Supplement: Supplementary file 1 — Appendix [file MSB-14-e8376-s001.pdf]

Appendix: Metabolic oscillations on the circadian time scale in *Drosophila* cells lacking clock genes

Guillaume Rey[1*#], Nikolay B. Milev[1#], Utham K. Valekunja[1], Ratnasekhar Ch[1,2], Sandipan Ray[1,2], Mariana Silva Dos Santos[1], Andras D. Nagy[1], Robin Antrobus[3], James I. MacRae[1], and Akhilesh B. Reddy[1*]

[1] The Francis Crick Institute, 1 Midland Road, London NW1 1AT, UK.
[2] UCL Institute of Neurology, Queen Square, London WC1N 3BG, UK.
[3] Cambridge Institute for Medical Research (CIMR), Wellcome Trust/MRC Building, Addenbrooke's Hospital, Cambridge CB2 0XY, UK.

Table of contents

Appendix Figure S1. RNA-Seq data analysis.

Appendix Figure S2. Cell cycle analyses in S2 cells.

Appendix Figure S3. RNA-Seq time course at 28°C and temperature compensation.

Appendix Figure S4. Proteomics data analysis.

Appendix Figure S5. Integration of transcriptomics and proteomics data.

Appendix Figure S6. Metabolomics data analysis.

Appendix Figure S7. Coupled gene expression and metabolic cycles in *Drosophila* S2 cells.

Appendix Table S1. Table of the 70 MS2-annotated features.

Appendix Table S2. Table of the 20 metabolites selected for targeted LC-MS analysis.

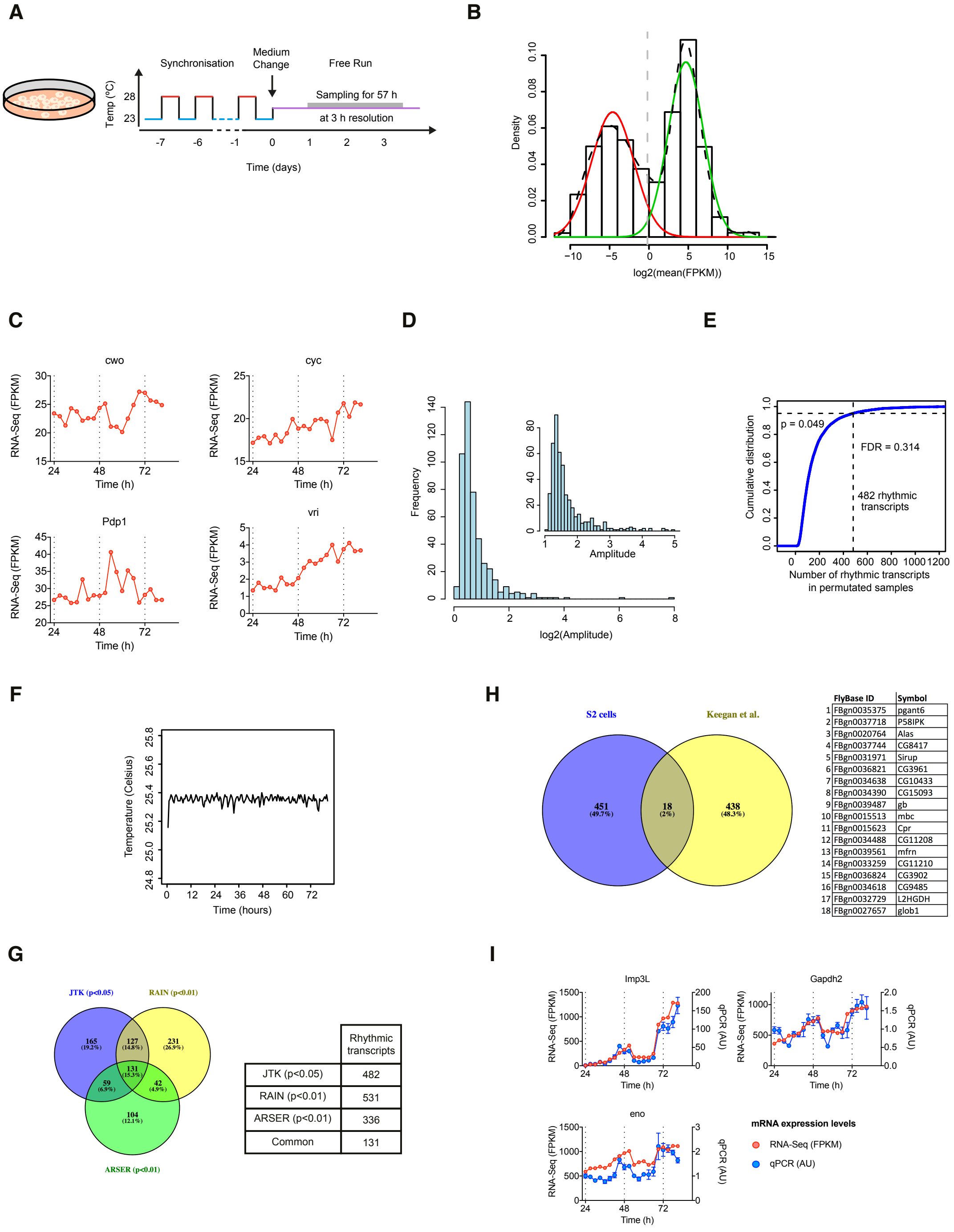

Appendix Figure S1. RNA-Seq data analysis.

A, Schematic showing the entrainment protocol used to synchronize *Drosophila* S2 cells. **B,** Histogram showing the distribution of mean expression levels measured by RNA-Seq. The distribution was modelled with a mixture model to define the sets of low (red) and highly (green) expressed transcripts. The dashed vertical line represents the cut-off chosen to define the set of expressed transcripts. FPKM, Fragments Per Kilobase of transcript per Million mapped reads. **C,** mRNA expression of four canonical circadian expressed in S2 cells. **D,** Distribution of amplitudes of

the 482 rhythmic transcripts. Amplitudes were calculated by taking the ratio between the maximum and the minimum of each transcript profile. Insert: zoomed version of the plot with a natural scale on the x-axis. **E,** Estimation of FDR using permutation tests. The cumulative distribution of the number of rhythmic transcripts in the 10,000 permutations is shown together with the empirical p-value and estimated FDR. **F,** Temperature recordings inside the incubator chamber show that the temperature is stable and not oscillating during the free-run phase. **G,** Comparison of the JTK-Cycle algorithm with two other methods to detect rhythmic transcripts. A table giving the number of rhythmic transcripts is given (left), together with a Venn diagram showing the overlap between methods (right). **H,** Overlap between the 482 rhythmic transcripts found in the study and the set of 456 rhythmic transcripts compiled from 5 microarray studies (Keegan *et al*, 2007). **I,** Validation of selected transcripts by quantitative PCR (qPCR) (n = 3, mean ± SEM).

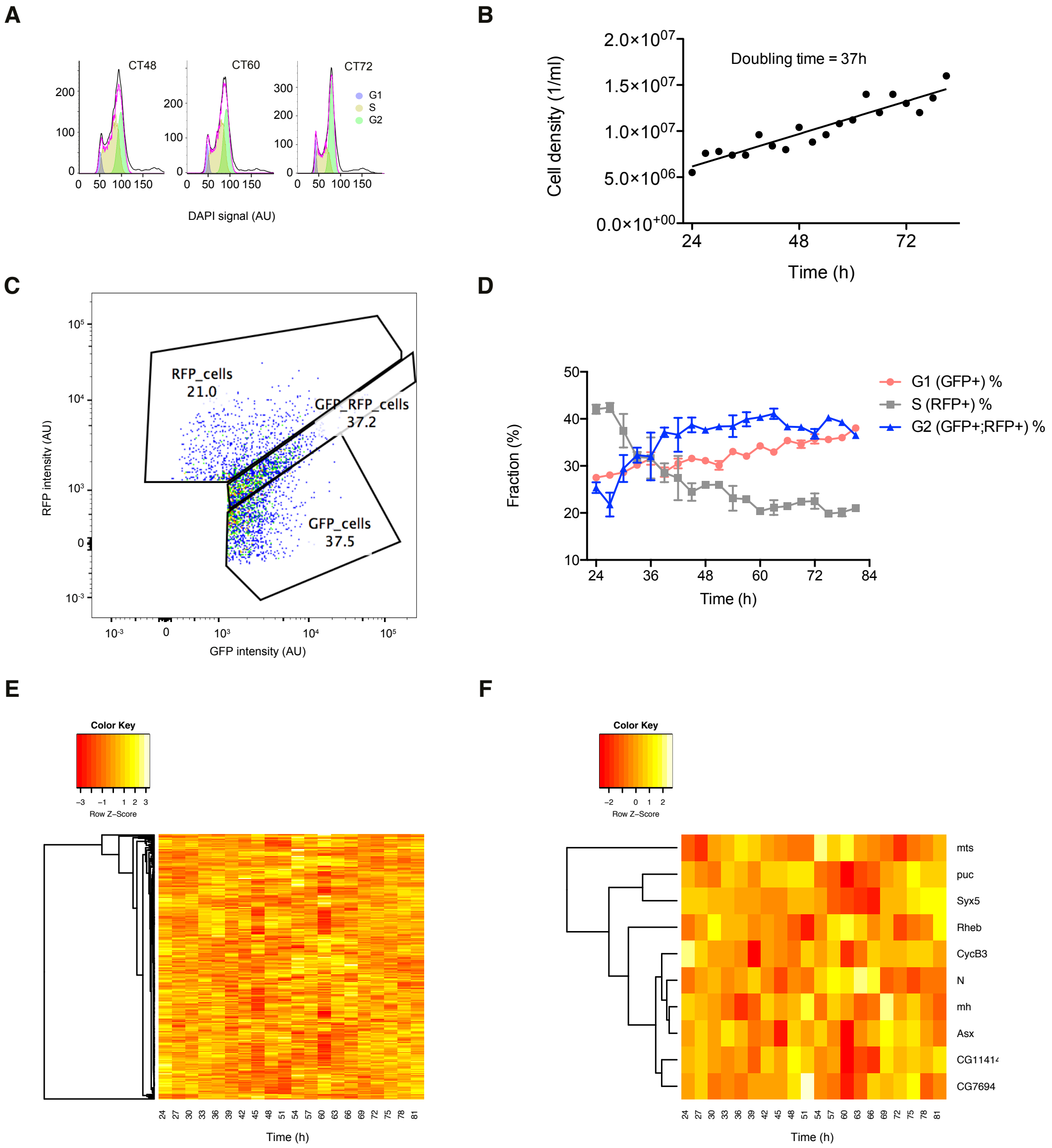

Appendix Figure S2. Cell cycle analyses in S2 cells.

A, Representative histograms of the DAPI signal at selected time points, with the curve fitting of cell cycle phases. **B,** Cell density of S2 cells during the time course experiment is shown, together with the estimated double time. **C,** Representative image from the flow cytometry analysis of Fly-FUCCI S2 cells showing the windows used to define GFP and RFP positive cells. **D,** Cell cycle analysis of Fly-FUCCI S2 cells using flow cytometry showing the fraction of cells in G1, S and G2 phases over a time course experiment (n = 3 per time-point, mean ± SEM). **E,** Heatmap representation of transcripts with mitotic cell cycle GO annotation from the 25ºC transcriptomics experiment. **F,** Heatmap representation of rhythmic transcripts with mitotic cell cycle GO annotation from the 25ºC transcriptomics experiment.

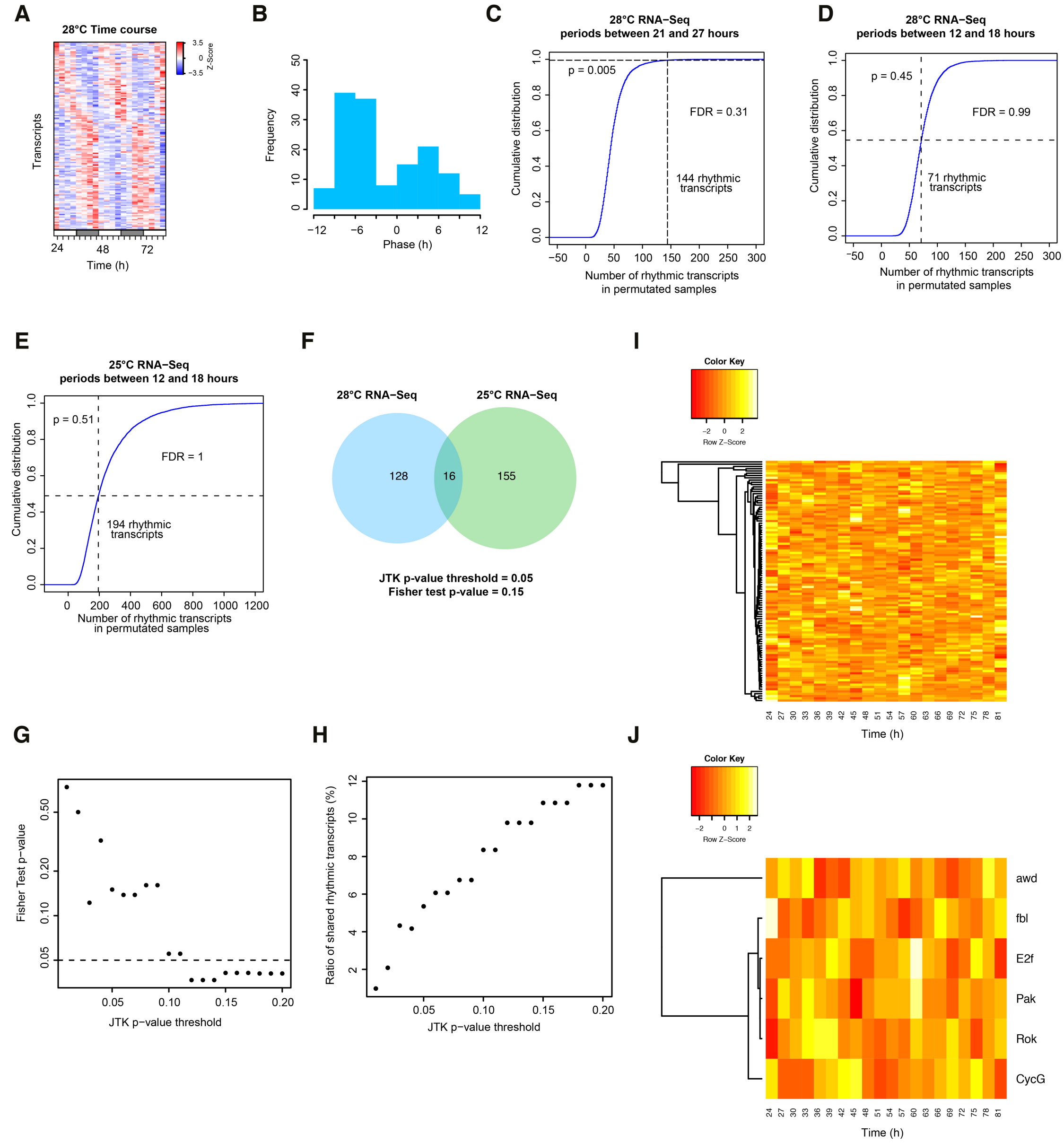

Appendix Figure S3. RNA-Seq time course at 28°C and temperature compensation.

A, Heatmap showing the expression profiles of the 144 rhythmic transcripts (JTK-Cycle, $p < 0.05$, FDR=0.31) **B,** Distribution of phase of the circadian transcripts shown in **A**. **C, D** Estimation of FDR using permutation tests for JTK analyses for periods of 21-27h (**C**) and 12-18h (**D**). The cumulative distribution of the number of rhythmic transcripts in the 10,000 permutations is shown together with the empirical p-value and estimated FDR. **E,** Estimation of FDR for JTK analyses for periods 12-18h in the 25°C experiment. **F,** Overlap between rhythmic transcripts detected at 25°C and 28°C shown with a Venn diagram. **G,** Statistical significance of the overlap between rhythmic transcripts detected at 25°C and 28°C as a function of the threshold of the JTK algorithm. **H,** Ratio of shared rhythmic transcripts detected at 25°C and 28°C, (# of shared rhythmic transcripts)/(# of rhythmic transcripts detected in either experiment), as a function of the threshold of the JTK algorithm. **I,** Heatmap representation of transcripts with mitotic cell cycle GO annotation from the 28ºC transcriptomics

experiment. **J,** Heatmap representation of rhythmic transcripts with mitotic cell cycle GO annotation from the 28ºC transcriptomics experiment.

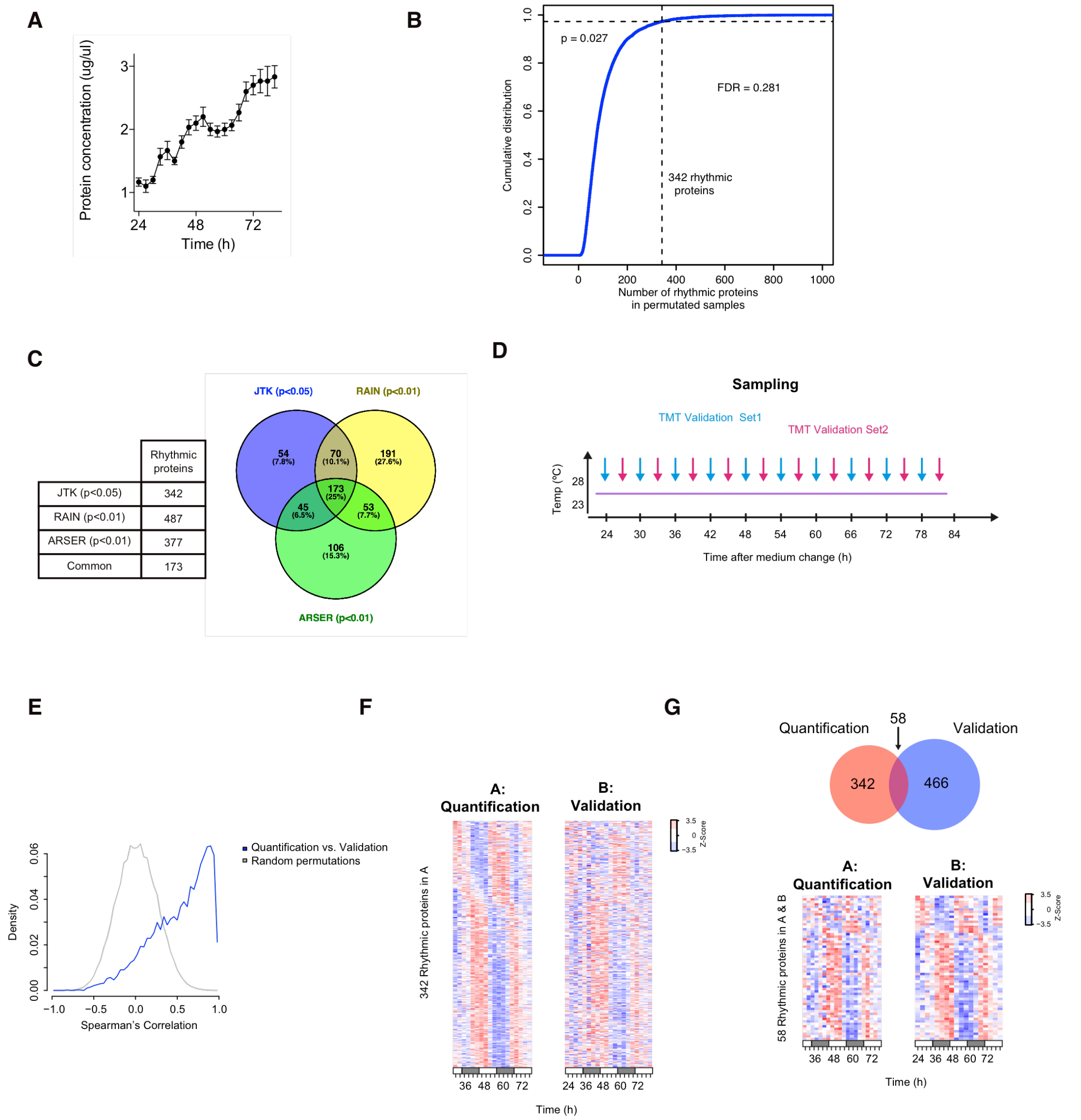

Appendix Figure S4. Proteomics data analysis.

A, Protein concentration in cell extracts as measured by the BCA method (mean ± SEM, n = 2-3). **B,** Estimation of FDR using permutation tests. The cumulative distribution of the number of rhythmic proteins in the 10,000 permutations is shown together with the empirical p-value and estimated FDR. **C,** Validation of the JTK-Cycle algorithm with two other methods to detect rhythmic transcripts. A table giving the number of rhythmic transcripts is given (left), together with a Venn diagram showing the overlap between methods (right). **D,** Cartoon representing the labelling scheme for the validation protocol. Samples were labelled using interspersed time points (Set 1: CT24, CT30, CT36, …; Set 2: CT27, CT33, CT39, …) and the two 10plex TMTs were then combined to produce 20 time point quantification profiles. **E,** Distributions of Spearman's correlation coefficient between the quantification and validation sets of quantitative proteomics data. Random permutations of the data are shown as control (n = 100). **F,** Heatmap representation of the proteins detected as rhythmic in the

quantification set (342 proteins) for the quantification set (left) and validation set (right). **G,** Heatmap representation of the proteins detected as rhythmic in both the quantification and validation sets (58 proteins) for the quantification set (left) and validation set (right).

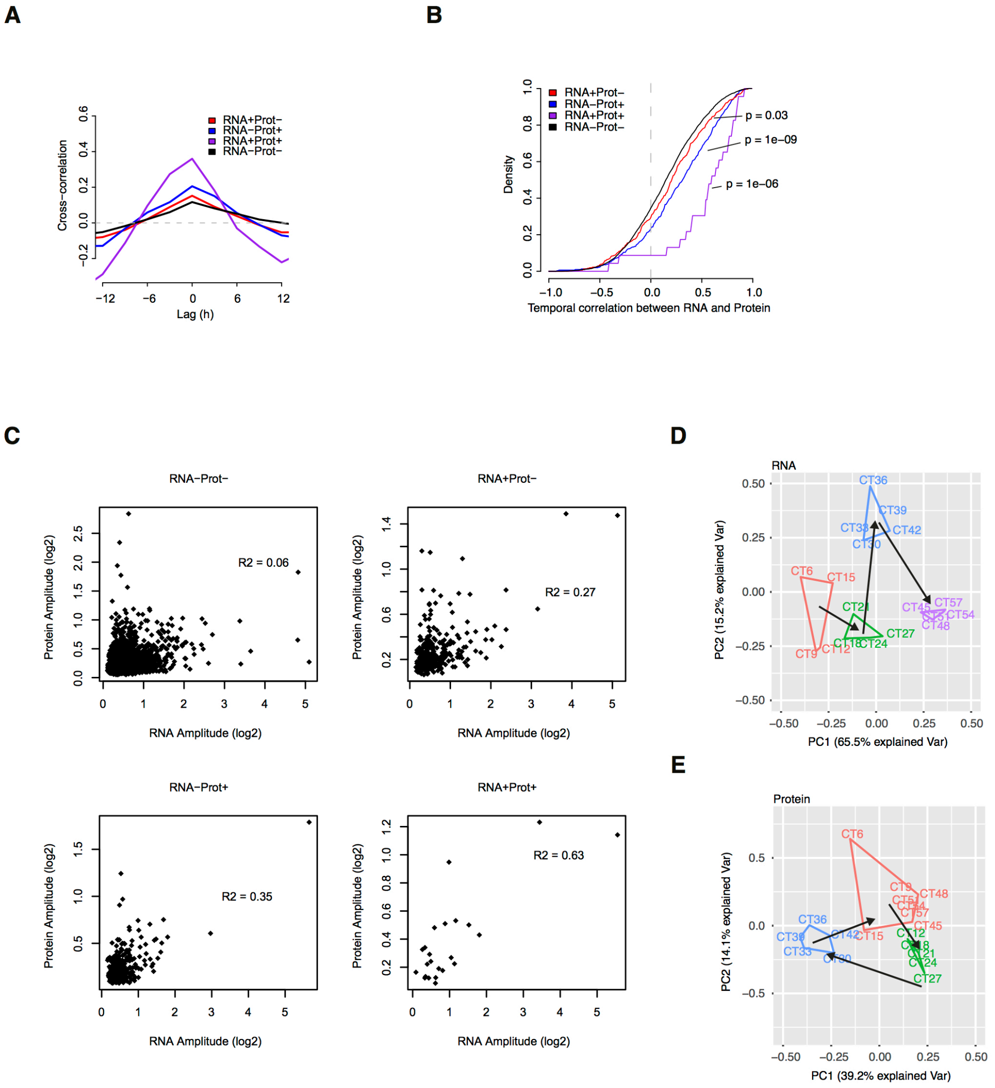

Appendix Figure S5. Integration of transcriptomics and proteomics data.

A, Temporal cross-correlation for RNA-proteins pairs as described in Figure 3. **B,** Cumulative distributions of Pearson correlation coefficients for the indicated groups of RNA-protein pairs for detrended profiles. RNA-Prot-, neither circadian; RNA+Prot+, both circadian; RNA-Prot+, RNA not circadian and protein circadian; RNA+Prot-, RNA circadian and protein not circadian (Wilcoxon sum rank test). **C,** Scatterplot of amplitude at the transcript and protein levels for the 4 sets of RNA-protein pairs. The coefficient of determination of the linear regression is shown for each comparison. **D, E,** PCA plot of RNA and protein data for all RNA-proteins pairs. Clustering of time points is performed with Partitioning Around Medoids (PAM) method.

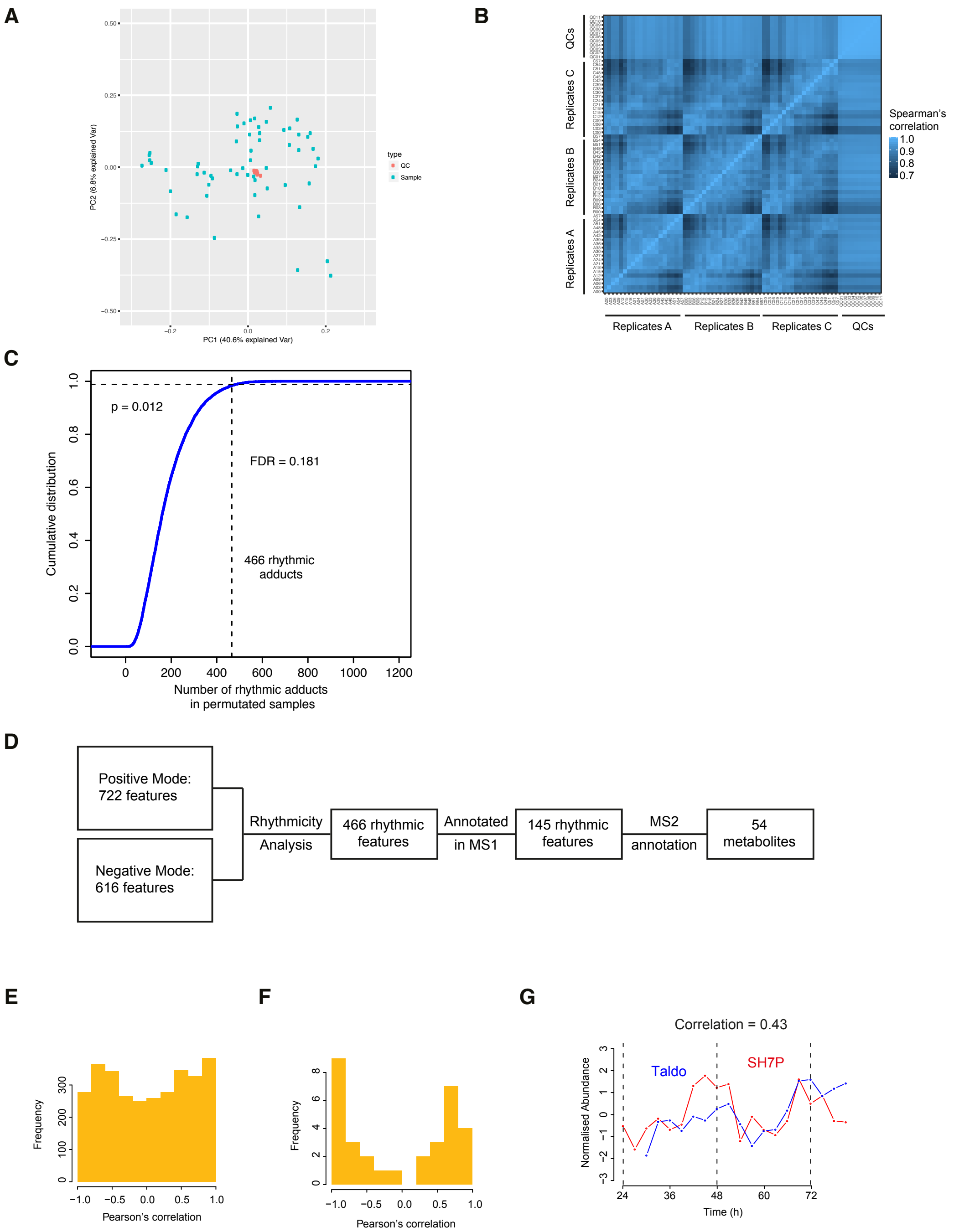

Appendix Figure S6. Metabolomics data analysis.

A, PCA plot of the 59 samples and 11 quality control (QC) samples analysed in this study. **B,** Heatmap showing the Spearman's correlation between all samples. **C,** Estimation of FDR using permutation tests. The cumulative distribution of the number of rhythmic features in the 10,000 permutations is shown together with the empirical p-value and estimated FDR. **D,** Workflow of untargeted metabolomics data analysis. **E,** Distribution of Pearson's correlation coefficients for all possible protein-metabolite pairs. **F,** Distribution of Pearson's correlation coefficients for the best protein-metabolite pairs. For each metabolite, the correlation coefficients for all possible interacting

proteins were computed and the protein with the highest absolute correlation coefficient was retained. **G,** Examples of correlated protein-metabolites are shown with Pearson correlation coefficient (top). SH7P, sedoheptulose 7-phosphate; Taldo, Transaldolase.

A

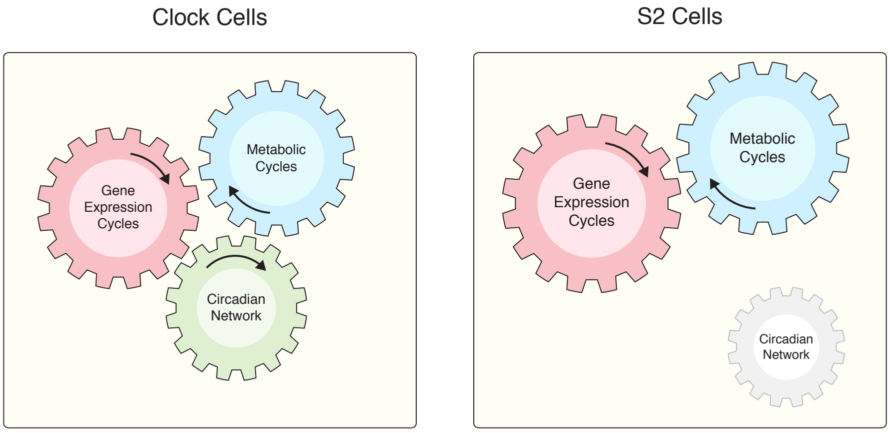

Appendix Figure S7. Coupled gene expression and metabolic cycles in *Drosophila* S2 cells. A, Schematic showing the difference between a clock cell where the circadian network is active and *Drosophila* S2 cells, which exhibit gene expression and metabolic cycles in its absence.

Appendix Table S1.

Table of the 70 MS2-annotated features with m/z, retention time and fragments used for annotation.

m/z	Retention time (min)	Charge	Polarity	MS2 Annotation	CAS	KEGG	Fragments
132.07	12.17	1	Positive	3-Hydroxy-L-proline	4298-08-2	C19706	90.06, 132.08
136.05	9.78	1	Positive	Adenine	73-24-5	C00147	119.04, 92.02, 136.06
508.00	12.85	1	Positive	Adenosine triphosphate	56-65-5	C00002	136.06, 410.03, 428.04
118.09	10.80	1	Positive	Betaine	107-43-7	C00719	118.09
332.14	8.15	1	Positive	Ciprofloxacin	85721-33-1	C05349	231.06, 332.14
489.11	12.79	1	Positive	Citicoline	987-78-0	C00307	264.04, 184.07
223.07	13.56	1	Positive	Cystathionine	535-34-2	C00542	134.08, 223.08
244.09	11.22	1	Positive	Cytidine	65-46-3	C00475	95.02, 112.05
324.06	13.27	1	Positive	cytidine 5-monophosphate	63-37-6	C00055	112.05, 324.06
112.05	13.27	1	Positive	Cytosine	71-30-7	C00380	95.02, 112.05
112.05	11.22	1	Positive	Cytosine	71-30-7	C00380	95.02, 112.05
148.06	12.68	1	Positive	Glutamic acid	56-86-0	C00025	84.04, 102.06, 130.05
156.08	16.29	1	Positive	Histidine	71-00-1	C00135	110.07, 113.11,156.08
127.07	11.18	1	Positive	Imizadoleacetic acid	645-65-8		81.04, 85.05
88.11	10.29	1	Positive	Isoamylamine	107-85-7	C02640	88.08, 70.07
170.09	7.42	1	Positive	Methyl-L-histidinate	1499-46-3		110.07, 170.09
174.11	7.10	1	Positive	N-Acetyl-L-leucine	1188-21-2	C02710	174.06
664.12	12.27	1	Positive	NAD	53-84-9	C00003	428.04, 136.06, 524.06
124.04	8.08	1	Positive	Niacin	59-67-6	C00253	124.04, 80.05, 96.04
166.09	10.11	1	Positive	Phenylalanine	63-91-2	C00079	120.08, 166.09
166.07	9.82	1	Positive	Phenylalanine	63-91-2	C00079	120.08
130.09	11.47	1	Positive	Pipecolic Acid	3105-95-1	C00408	84.08, 130.09
218.14	9.86	1	Positive	propionylcarnitine	17298-37-2	C03017	85.03, 218.14, 159.07
377.15	8.81	1	Positive	Riboflavin	83-88-5	C00255	243.09
399.14	12.95	1	Positive	S-Adenosyl-L-methionine	29908-03-0	C00019	250.09, 298.10, 136.06
106.05	13.17	1	Positive	Serine	56-45-1	C00065	60.04
203.15	14.66	1	Positive	Symmetric Dimethylarginine	30344-00-4		203.15, 116.07, 70.07, 89.09
150.11	8.86	1	Positive	Triethanolamine	102-71-6	C06771	70.07, 88.08, 132.10
183.09	12.33	1	Positive	Triethyl phosphate	78-40-0		98.98, 127.02
182.08	11.89	1	Positive	Tyrosine	60-18-4	C00082	136.08, 123.04, 119.05, 91.05
296.08	7.13	1	Negative	5 -S- methyl -5- thioadenosine	2457-80-9	C00170	134.05
130.05	11.24	1	Negative	5-aminolevulinic acid	106-60-5	C00430	130.09
173.01	13.68	1	Negative	Aconitic Acid	585-84-2	C00417	85.03, 111.01
505.99	12.85	1	Negative	Adenosine triphosphate	56-65-5	C00002	505.99, 158.92, 408.01, 176.93
88.04	12.70	1	Negative	Alanine	302-72-7	C01401	88.04
289.12	13.45	1	Negative	Argininosuccinic acid	2387-71-5	C03406	131.08, 132.03, 173.1
132.03	12.85	1	Negative	Aspartic acid	56-84-8	C00049	88.04, 71.01, 72.01
191.02	13.75	1	Negative	Citric acid	77-92-9	C00158	87.01, 111.01, 85.03
221.06	13.56	1	Negative	Cystathionine	535-34-2	C00542	134.03, 120.01, 221.06
147.03	12.76	1	Negative	D-alpha-Hydroxyglutaric acid	2889-31-8	C01087	85.03, 101.02, 57.03, 103.04, 129.02
195.05	12.41	1	Negative	D-Gluconic acid	526-95-4	C00257	75.01, 195.05, 129.02
171.10	7.09	1	Negative	Decanoic acid	334-48-5	C01571	171.14
171.10	4.44	1	Negative	Decanoic acid	334-48-5	C01571	171.14
171.14	4.16	1	Negative	Decanoic acid	334-48-5	C01571	171.14
121.05	11.20	1	Negative	Erythritol	149-32-6	C00503	71.01, 121.03, 89.02
259.02	13.43	1	Negative	Glucose 6-phosphate	56-73-5	C00092	96.97, 78.96
129.02	12.76	1	Negative	Glutaconic acid	1724-02-3	C02214	85.03, 101.02
146.05	12.70	1	Negative	Glutamic acid	56-86-0	C00025	102.06, 128.03, 146.05
145.06	12.78	1	Negative	Glutamine	5959-95-5	C00819	101.02, 145.06, 127.05
152.53	12.46	2	Negative	Glutathione	70-18-8	C00051	74.02, 128.03, 152.87, 86.02
171.01	12.68	1	Negative	Glycerol 3-phosphate	57-03-4	C03189	78.96, 96.97
171.08	11.65	1	Negative	Glycylproline	704-15-4		171.14, 96.96
521.98	13.97	1	Negative	Guanosine triphosphate	86-01-1	C00044	521.98, 424.00
154.06	16.03	1	Negative	Histidine	150-35-6	C00135	154.06, 137.03, 93.04
103.00	13.03	1	Negative	Malonic acid	141-82-2	C00383	59.01, 73.03
181.07	13.89	1	Negative	Mannitol	69-65-8	C00392	181.07, 101.02, 71.01
181.07	12.93	1	Negative	Mannitol	69-65-8	C00392	181.07, 101.02, 71.01
181.07	14.25	1	Negative	Mannitol	69-65-8	C00392	181.07, 101.02, 71.01
172.10	4.67	1	Negative	N-Acetyl-L-leucine	1188-21-2	C02710	172.14, 130.09
190.05	4.93	1	Negative	N-Acetyl-L-methionine	65-82-7	C02712	84.04
190.05	7.13	1	Negative	N-Acetyl-L-methionine	65-82-8	C02713	84.04
122.02	8.09	1	Negative	Nicotinic acid	59-67-6	C00253	94.03, 122.02, 78.03
164.07	10.12	1	Negative	Phenylalanine	63-91-2	C00079	147.04, 164.07, 72.01
166.97	13.49	1	Negative	Phosphoenolpyruvic acid	138-38-9	C00074	78.96
128.07	11.50	1	Negative	Pipecolic acid	535-75-1	C00408	128.03
117.02	12.76	1	Negative	Succinic acid	110-15-6	C00042	73.03, 117.02, 99.01
203.08	11.08	1	Negative	Tryptophan	54-12-6	C00806	203.08, 116.05, 159.09
180.07	11.87	1	Negative	Tyrosine	60-18-4	C00082	180.07, 163.04, 119.05
167.02	11.87	1	Negative	Uric Acid	13154-20-6	C00366	69.01, 124.01, 96.02
151.03	11.20	1	Negative	Xanthine	69-89-6	C00385	151.03, 108.02, 136.02

Appendix Table S2.

Table of the 20 metabolites selected for targeted LC-MS analysis.

m/z	Polarity	RT Start (min)	RT End (min)	Name	Abbreviation
261.037	Positive	12.8	13.8	Glucose 6-phosphate	G6P
308.0911	Positive	11.8	12.8	Glutathione reduced	GSH
348.07036	Positive	11.5	12.5	Adenosine monophosphate	AMP
428.0367	Positive	11.9	12.9	Adenosine diphosphate	ADP
508.00304	Positive	12.4	13.4	Adenosine triphosphate	ATP
613.1593	Positive	12.8	13.8	Glutathione oxidized	GSSG
664.11641	Positive	11.1	12.1	Nicotinamide adenine dinucleotide	NAD
744.08272	Positive	12.4	13.4	Nicotinamide adenine dinucleotide phosphate	NADP
89.02441	Negative	9.5	10.5	Lactic acid	Lac
115.0037	Negative	12.5	13.5	Fumaric acid	Fum
117.01933	Negative	12.2	13.2	Succinic acid	Succ
133.0143	Negative	12.5	13.5	Malic acid	Mal
145.0143	Negative	12.1	13.1	Alphaketoglutaric acid	a-KG
166.9751	Negative	12.9	13.9	Phosphoenolpyruvic acid	PEP
168.9908	Negative	12.2	13.2	DHAP	DHAP
191.0197	Negative	13.1	14.1	(Iso)citrate	(Iso)cit
229.0119	Negative	12.4	13.4	Ribose 5-phosphate/Ribulose 5-phosphate	R5P/Ru5P
259.0224	Negative	12.8	13.8	Fructose 6-phosphate	F6P
289.033	Negative	12.5	13.5	Sedoheptulose 7-phosphsate	SH7P
338.9888	Negative	13	14	Fructose 1,6-biphosphate	FBP
